# A Quality Improvement Initiative: Supporting the Potential of Foundation Year Doctors with a "Trauma Innovation Forum"

**DOI:** 10.7759/cureus.7705

**Published:** 2020-04-17

**Authors:** Gregory Neal-Smith, David S Kim, Henry A Claireaux, Alexander Wood

**Affiliations:** 1 Trauma and Orthopaedics, Oxford University Hospitals NHS Foundation Trust, Oxford, GBR; 2 Trauma and Orthopaedics, Oxford University Medical School, Oxford, GBR; 3 Orthopaedics, Oxford Trauma, Oxford, GBR; 4 Trauma, Oxford University Hospitals NHS Foundation Trust, Oxford, GBR

**Keywords:** quality improvement, multidisciplinary team, foundation doctors, innovation forum, reproducibility, protected environment, constructive criticism, nurse practitioners, trauma and orthopaedics, patient care

## Abstract

Junior doctors rotating through departments arrive with fresh perspectives and are particularly suited for identifying problems and providing creative solutions to improve patient care. They may, however, be unfamiliar with the process of implementing an idea into practice. We recognize the need to support foundation year doctors to develop successful quality improvement projects (QIPs). We developed a new initiative to host an annual event that gives foundation year doctors a platform to develop QIPs for their department. These ideas were pitched to an audience comprising trauma consultants from the Oxford University Hospitals and multidisciplinary staff from hospitals across the region. It offered a dedicated time away from clinical pressure to propose and receive immediate expert feedback from members of the trauma multidisciplinary team. With this refinement and supervisor project matching, it facilitated multiple areas of targeted change for the department in just one evening. In total, eight QIPs were developed from the event. Here we outline our approach and the structure of our event, which can serve as a tool for similar initiatives to be replicated in other hospitals.

## Introduction

Foundation year doctors offer a unique perspective on the healthcare service. This title is given to doctors in their first two years of work in the United Kingdom's National Health Service after graduation from medical school. Their roles include rotations through multiple wards, specialties, and hospitals within their allocated region. This enables them to experience a range of different protocols and standard operating procedures unique to each working environment. Their extensive contact time with patients, nurses, and hospital administrative systems provides the individual with a different insight compared with their senior colleagues. This is an asset to any department and can be utilized if they are presented with the right opportunities to implement meaningful changes to the service.

Recent literature has suggested that quality improvement is better with a bottom-up approach, whereby the motivations of clinical staff are harnessed to drive improvement [1]. Other studies have reported that junior doctors are underutilized in patient safety and quality improvement projects [2]. This involvement also leads to more sustainable quality improvement cultures that can be embedded within organizations [1]. Particularly, key is improvement in the field of trauma and orthopedic surgery, where national database evidence has highlighted concerns over the level of iatrogenic harm [3]. The largest proportion of surgical patient safety incidents were reported from the trauma and orthopedic specialty, with most errors relating to the implementation of care and on-going patient monitoring [3]. It is for these reasons that foundation year doctors are particularly apt at targeting improvements in patient care, communication across the multidisciplinary team (MDT), local pathways, and IT systems.

Here we outline the structure of a successful initiative: the "Trauma Innovation Forum". We highlight the following four criteria proposed by the event organizing team to develop effective QIPs:

1. Need for constructive criticism and input of other perspectives to evaluate the feasibility of implementing an idea.

2. Opportunities for doctors to present ideas to key individuals who are local to the department.

3. Need for appropriate support and backing from the relevant consultants within the specialty.

4) Need for staff at all levels of training to engage and openly discuss key issues faced when delivering care.

The event aimed to meet the preceding four criteria. It facilitated the proposal and discussion of multiple QIPs in an open and constructive environment without the immediate clinical pressures of being on shift. Key issues raised by foundation year doctors were addressed with feedback sources such as senior consultants, nurse practitioners, and registrars from multiple trauma departments within the region. The event enabled a number of consultants to select and supervise QIP proposals of their interest, which benefited both the foundation doctors and the consultants. This was crucial in assisting several projects to go on to seek funding or initiate implementation within the department.

## Materials and methods

The "Trauma Innovation Forum" event followed by a formal dinner was organized, with audience comprising consultants, registrars, nurse practitioners, and foundation year doctors. The event started with an educational presentation from a medical technology company before the innovation presentations and discussions began.

All foundation year doctors who had rotated through the trauma department in the last year had been contacted the previous month with an invitation to attend the event. They were asked to give a 10-minute presentation on an idea that would improve the quality of care delivered to patients in the department. These ranged from implementing point-of-care imaging in triage to improving documentation in handovers. The audience were then consulted at the end of each presentation to scrutinize and challenge the ideas presented.

A selected panel of trauma consultants and nurse practitioners were then asked to individually score each presentation based on the following criteria:

1. Identifying a problem.

2. Offering solutions.

3. Evidence of solution.

4. Feasibility of solution.

5. Presentation of solution to the audience.

The judges then gathered following the discussions to select the highest scoring presentations. Prizes were offered at the end to the QIPs with the highest score.

Trauma consultants were then matched to the individual presenters of the QIPs based on their interest so that they could discuss the implementation and future steps. All the projects were then raised by the consultants at the surgical departmental meetings to notify all members of the MDT of the changes.

## Results

Opportunity to present ideas

The proposals from the foundation year doctors covered a wide variety of departmental topics and issues. In total, eight unique QIPs were developed from the event, which are as follows:

1. Introduction of ultrasound to improve the diagnostic pathway in acute knee injury.

2. Digital improvement in a major trauma center.

3. Fascia iliaca block training for junior doctors.

4. Improving access of the operating theater to junior doctors in a major trauma center.

5. Changing the induction process for trainees in the trauma department.

6. Exploring new digital initiatives in the trauma department.

7. A new handover protocol for morning and night handover meetings.

8. Improving junior involvement in major trauma research.

Feedback

Following the event, a survey was sent out to the foundation year doctors who proposed their QIPs, with a response rate of 91% (10/11). Our findings indicate that:

1. 100% (10/10) of participants felt more encouraged to present their ideas to their colleagues in the future after the event.

2. 100% (10/10) of participants found constructive criticism and audience engagement helpful in refining their QIP.

3. 100% (10/10) of participants felt that this event was a good way of raising issues and developing solutions within a department.

The foundation year doctors were asked to rate the event overall on a scale of 5. The average score for the event was 4.9, which demonstrates that this is a successful method of providing foundation years doctors a platform to speak up and empower them to improve departments.

## Discussion

The need for criticism

The opportunity to present these ideas at an early stage to the MDT enabled both the feasibility and potential impact of the QIP to be estimated through a diverse range of professional perspectives within the department. By engaging with staff across various healthcare roles, any major pitfalls of implementing the QIPs were flagged, thus acting as an immediate filter. The presenters were able to gauge the contextual validity of their proposal through audience engagement. Various colleagues offered their insights from their own experiences to challenge aspects of its feasibility. Through this, they were able to suggest amendments and counter-proposals that formulated an improved version of each QIP to the ones originally presented. It was also helpful having representatives from other hospitals in the region, as they were able to share ideas and discuss their own local protocols. Through this method of gaining perspectives from both senior colleagues and peers, we firstly enabled ideas to be refined into more workable solutions, and, secondly, enhanced the credibility of each QIP proposal for future grant applications and clinical impact.

The need for appropriate support

The event enabled junior doctors with new perspectives to pitch their ideas and find support from relevant consultants and registrars. With direction from more senior colleagues, several ideas that emerged from this event have since obtained grants for equipment, begun local trials, or been fully implemented within the Oxford University Hospitals.

The need for staff engagement

Organizing opportunities such as this, which facilitated meaningful discussion with a central focus on improving patient care, is extremely beneficial to departments. Foundation year doctors were encouraged by their senior colleagues to think broadly about challenging the current protocols with their insights, which promoted an ethos of innovation.

Limitations

This study has reported the advantages of utilizing foundation year doctors and providing them a platform to facilitate QIP development; however, there are limitations as well. Foundation year doctors have shorter placements and frequently rotate specialties, which can make it difficult for them to complete full QIP cycles. On top of this, their experience of designing QIPs is often more limited than their senior colleagues. That is the benefit, however, of offering an event with constructive feedback - MDT discussion and pairing of supervisors simultaneously.

## Conclusions

The inaugural "Trauma Innovation Forum" was found to be a successful platform to utilize the unique perspectives of foundation year doctors to improve the local trauma department. Attendees from other trauma departments in the region provided insights into other hospital protocols and allowed the sharing of ideas. The event offered a dedicated time away from clinical pressures to propose and receive immediate expert feedback from members of the trauma MDT. With this refinement and supervisor project matching, it facilitated multiple areas of targeted change for the department in just one event. Finally, initiatives such as these promote confidence in foundation year doctors to present their ideas and encourage an ethos aimed at providing the best possible care for our patients. We hope that this approach and the structure of our event may serve as a tool for similar initiatives to be replicated in other hospitals.
